# Long non-coding RNA HOXB-AS1 is a prognostic marker and promotes hepatocellular carcinoma cells’ proliferation and invasion

**DOI:** 10.1515/biol-2022-0040

**Published:** 2022-08-15

**Authors:** Yubin Chen, Na Wang, Liangqi Cao, Dawei Zhang, Heping Peng, Ping Xue

**Affiliations:** Department of Hepatobiliary Surgery, The Second Affiliated Hospital of Guangzhou Medical University, No. 63 Yayun South Road, Guangzhou, 510000, Guangdong Province, China

**Keywords:** HOXB-AS1, hepatocellular carcinoma, Hep3B, Huh7

## Abstract

Long non-coding RNAs (lncRNAs) are broadly transcribed in the genome of human and play critical roles in the progression of multiple diseases. Long non-coding HOXB cluster antisense RNA 1 (HOXB-AS1) is a tumor exciter in various cancers. This study aimed to investigate the involvement of HOXB-AS1 in hepatocellular carcinoma (HCC). In the following study, HOXB-AS1 was unveiled to be highly expressed in HCC tissues as opposed to normal tissues. Silencing of HOXB-AS1 led to the loss of proliferation, migration, and invasiveness of HCC cells, namely Hep3B and Huh7. Moreover, the data showed that expression levels of HOXB-AS1 contribute significantly to the patient’s survival rates. Otherwise, HOXB-AS1 levels in the serum of patients proved HOXB-AS1 as a biomarker for analysis and treatment of HCC. In summary, this study highlights HOXB-AS1 as key upregulated lncRNA in HCC which being an oncogene can cause proliferation and metastasis of HCC cells. The results also highlighted HOXB-AS1 as a promising biomarker for early diagnosis and prognosis of patients with HCC.

## 1 Introduction

Hepatocellular carcinoma (HCC) has become the most common subtype of liver cancer in the last decade, comprising 90% of primary liver cancers [1]. Clinical studies of HCC patients have demonstrated very low survival rates, accounting for more than 600,000 global deaths [2]. However, many information gaps elaborating the exact molecular mechanisms, protein alterations, and hereditary factors exist and need to be further investigated.

Long non-coding RNAs (lncRNAs) with >200 nucleotides have been demonstrated for their vital roles in physiological, developmental, and differential biological processes [3] in human diseases, particularly carcinogenesis [4,5]. LncRNAs can affect the normal molecular processes by binding different transcription factors, localizing cellular proteins to specific sites, facilitating the intermolecular interaction of various components, and differentially controlling and monitoring the expression of different genes at a particular stage in a tissue-specific manner [6,7]. Several transcriptome sequence platforms and microarray techniques have highlighted the abnormal and elevated levels of lncRNAs in HCC angiogenesis, metastasis, apoptosis, progression, and proliferation [8]. For example, Tsang et al. [9] showed aberrant expression of lncRNAs in 20 pairs of HCCs and respective nontumorous liver tissues. Likewise, lncRNA HULC performs an auto-regulatory loop with miR-372 causing the blockade of translational repression and leads to overexpressed HULC in HCC [10]. MALAT-1 interferes with splicing regulation of diverse pre-mRNAs and contributes to metastasis of HCC [11]. Likewise, lncRNA HEIH causes cell cycle arrest by inhibiting the expression of the p16 gene [12]. Similarly, overexpressed intergenic lncRNA HOTAIR has been involved in HCC metastasis [13]. HOXB-AS1 is a significant lncRNA which has been evaluated for its oncogenic role in multiple myeloma, endometrial cancer, and glioma [14,15,16]. Nonetheless, its potential in HCC is still unknown. Therefore, this study concentrated on deciphering the role of HOXB-AS1 in HCC invasion and proliferation. From the HCC samples, we identified that HOXB-AS1 is significantly upregulated. We further characterized the role of HOXB-AS1 in HCC development and potential as a prognostic marker.

## 2 Materials and methods

### 2.1 Tissue samples

In total, 60 subjects with HCC tissues and matched adjacent normal liver tissues were listed. Patients who underwent surgical procedures in the affiliated hospital were chosen for the study. Following surgery, tumor tissues were immediately subjected to snap freeze in liquid nitrogen for subsequent experimental and analytical work.


**Informed consent:** Informed consent has been obtained from all individuals included in this study.
**Ethical approval:** The research related to human use has been complied with all the relevant national regulations, institutional policies, and in accordance with the tenets of the Helsinki Declaration, and has been approved by the authors’ institutional review board or equivalent committee.

### 2.2 Cell lines and transfection experiment

Four human cell lines, including Huh7, BEL-7404, HCCLM3, and Hep3B, and human normal liver cells L02 were chosen for the analysis. Cell lines were procured from Cell Bank of Chinese Academy of Science (Shanghai) and subjected to resuspension in a cell culture medium containing Dulbecco’s modified Eagle’s medium (90%) and fetal bovine serum (10%; R&D Systems, USA). Typical cell culture conditions comprising 37°C, 95% humidity, and 5% CO_2_ were maintained in an incubator [17]. Standard transfection was performed in pcDNA3.1, showing respect to HOXB-AS1 and HOXB-AS1 siRNAs, with a negative controls procured from RiboBio (China). Lipofectamine (LFN) 3000 reagent (Invitrogen, USA) was used for the transfection assay following the manufacturer’s guidelines, and transfection efficiency was measured by qRT-PCR [18].

### 2.3 Extraction of RNA and qRT-PCR for quantitative gene expression

Total tissue RNA was retrieved with Trizol reagent (A33250, Invitrogen), focusing on the manufacturer’s guidelines. RNA was quantified with a spectrophotometer (Thermo Scientific). First Strand cDNA Synthesis Kit (Roche Life Sciences, Germany) was utilized to synthesize RNA into cDNA according to the reference guidebook. A qScript One-Step RT-qPCR kit (95057-050, Quantabio, Beverly, MA, USA) was utilized to perform qRT-PCR in PCR system (LineGene, Latvia). Primer set for HOXB-AS1: F: 5′-GGGGACTCCAGCGAAAT-3′; R: 5′-ACCCGAAGCCCAACCAC-3′; U6: F: 5′-CTCGCTTCGGCAGCACA-3′; R: 5′-AACGCTTCACGAATTTGCGT-3′; GAPDH: F: 5′-CCCACTCCTCCACCTTTGAC-3′; R: 5′-CATACCAGGAAATGAGCTTGACAA-3′ were used according to reference publication. GAPDH and U6 were used as internal controls following the analysis of fold changes in quantitative gene expression through the 2^−ΔΔCT^ calculation method [19].

### 2.4 Cell counting kit-8 (CCK-8) bioassay to calculate cell proliferation

Cell viability was confirmed with the CCK-8 (Sigma-Aldrich) following the manufacturer’s reference book. Cells were plated into 96-well plates (3 × 10^3^ cells/well) and incubated for 0, 24, 48, 72, and 96 h. Following this, 10 µL of CCK-8 proliferation solution was subsequently added to each well at each point and further incubated for 1 h at 37°C. Cellular optical densities were measured at 450 nm by using a microplate reader (Bio-Rad, USA) [18].

### 2.5 Transwell assay to estimate cell migration and invasion

The migratory ability of the cells was assessed as narrated by Zhang et al. [20] by using a Transwell chamber with inserts of 8 mm pore sizes (Millipore, USA). Membranes without any coating were utilized for migration assay, whereas cell invasion was calculated with Matrigel-coated membranes. Briefly, 5 × 10^4^ Huh7 and Hep3B cells containing DMEM (250 μL) were planted into the upper chamber following the addition of FBS (10%, R&D Systems, USA) into the lower chamber. Incubation was performed at 37°C for 36 h, and the remaining cells were removed gently with the help of a cotton swab. Invasive or migrating Huh7 and Hep3B cells moved to the low chamber, adhered to methanol (20 min) and subsequently stained with 0.1% CV (R&D Systems, USA). Following staining for 20 min, imaging was done with the help of a phase-contrast microscope, choosing five fields of vision (Olympus, Japan).

### 2.6 Statistical analysis

Experiments were conducted in three sets and presented as mean value ± deviations from the standard value (SD). The significant difference was computed by using SPSS 23.0 followed by Dunnett’s test, ANOVA, and Pearson’s Correlation test. Clinicopathological characteristics of the patients were analyzed using the chi-square test, whereas survival rates were determined with the Kaplan–Meier survival curve. Unless otherwise indicated, the data are presented as the mean value ± SD. *p < 0.05 was considered statistically significant.

## 3 Results

### 3.1 HOXB-AS1 is highly expressed in HCC tissues and cells

**Figure 1:**
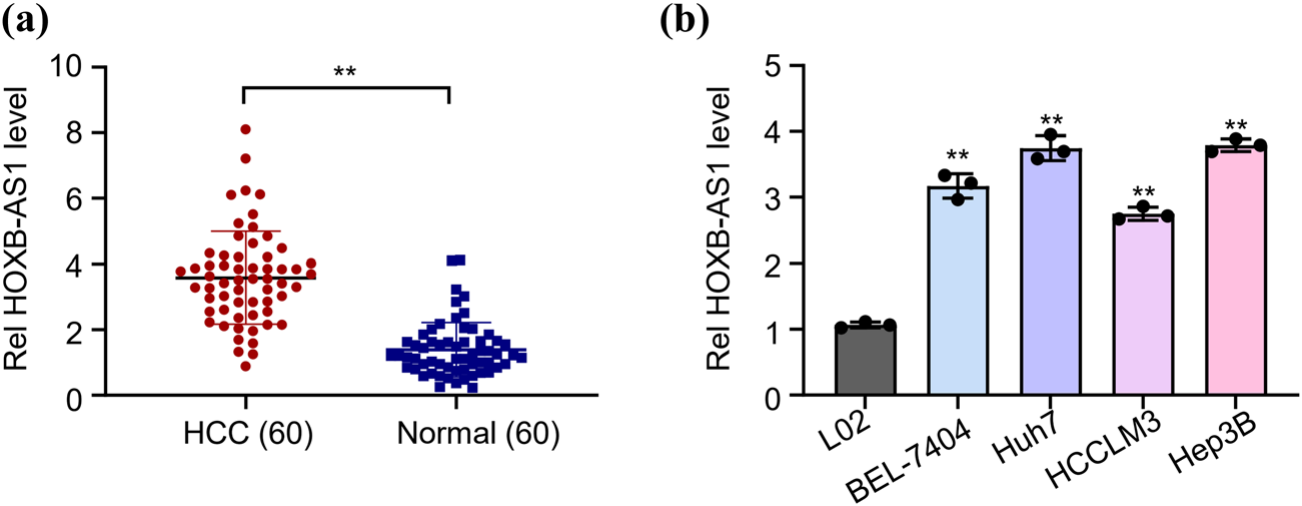
HOXB-AS1 is highly expressed in liver cancer tissues and cells. (a) HOXB-AS1 was significantly higher in HCC than in adjacent normal tissue. (b) The expression of HOXB-AS1 was increased in HCC cancer cell lines, namely Huh7, Hep3B, HCCLM3, and BEL7404. The results are representative of 3 separate experiments. **p < 0.01 vs normal group or L02 cell lines.

The study was designed to estimate and analyze the high and low expression levels of HOXB-AS1 in HCC, and for this, 60 pairs of HCC tissues and corresponding healthy tissues were put to qRT-PCR. The results from qRT-PCR clearly showed highly upregulated levels of HOXB-AS1 (**p < 0.01) in HCC, as shown in Figure 1a. To get a clear picture, patients were compared for the correlation of their clinicopathological characteristics and corresponding HOXB-AS1 levels. Considering the median expression value of HOXB-AS1 in HCC tissue as a cut-off value, 60 HCC patients were subdivided into two categories, one as exalted expression (*n* = 30) and the other as low expression (*n* = 30) HOXB-AS1 group. Results of chi-square analysis demonstrated the significance (**p < 0.001) and a direct correlation between lymph node and distant metastasis, TNM stage, and tumor differentiation. Nevertheless, no positive correlation was observed for HOXB-AS1 expression between the tumor size and localization, age, and sex of the patients (Table 1). Next the expression levels of HOXB-AS1 were analyzed in HCC cell lines, including Huh7, BEL-7404, HCCLM3, and Hep3B, and normal cell line L02 through qRT-PCR analysis. The results from PCR demonstrated highly expressed HOXB-AS1 levels in HCC cell lines as opposed to normal cells (**p < 0.01, Figure 1b). The highest expression of HOXB-AS1 was detected in Huh7 followed by Hep3B. Based on the highest correlated values, Huh7 and Hep3B were subjected to further analysis.

**Table 1:** The correlation between lncRNA HOXB-AS1 expression and clinicopathological variables of hepatocellular carcinoma patients

Clinicopathological characteristics	Total	HOXB-AS1 high expression (*n* = 30)	HOXB-AS1 low expression (*n* = 30)	*X* ^2^	*p* value
**Gender**					
Male	31	17	14	0.601	0.438
Female	29	13	16		
**Age**					
≤50	23	10	13	0.635	0.426
>50	37	20	17		
**Tumor size**					
T1 + T2	32	12	20	4.286	0.038
T3 + T4	28	18	10		
**Differentiation**					
High	19	15	4	10.850	0.004
Moderate	14	7	7		
Poor	17	8	19		
**Lymph node metastasis**					
Positive	26	18	8	6.787	0.009
Negative	34	12	22		
**TMN stages**					
I + II	25	7	18	8.297	0.004
III + IV	35	23	12		
**Distant metastasis**					
Yes		18	8	6.787	0.009
No		12	22		

### 3.2 Upregulated expression of HOXB-AS1 results in poor prognosis in HCC patients

**Figure 2:**
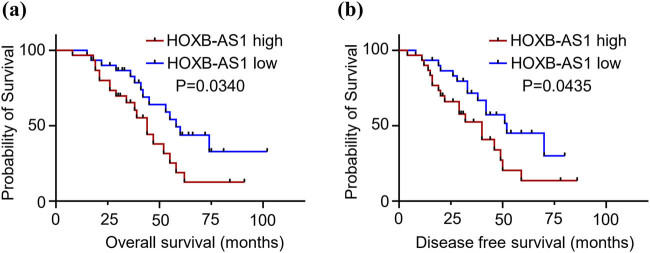
Upregulated expression of HOXB-AS1 results in poor prognosis in HCC patients. (a) Kaplan–Meier survival analysis of HOXB-AS1 high and low expression levels. (b) The probability of survival in the disease-free survival time of HCC patients in low and high HOXB-AS1 expression.

Next the survival rate for HCC patients with upregulated HOXB-AS1 was determined by using the Kaplan–Meier survival curve. Patients from high HOXB-AS1 expression group (*n* = 30) were compared with the individuals (*n* = 30) showing low expression profiles of HOXB-AS1. Results from the data comparison suggested overall survival of the individuals with high expression values of HOXB-AS1 was significantly less (*p* = 0.0340) as compared to subjects showing less expressed values of HOXB-AS1 (Figure 2a).

Following this, the probability of survival was used to evaluate the disease-free survival time of HCC patients in the low expression group (*n* = 30) and high expression group (*n* = 30) using the Kaplan–Meier survival curve. The results were similar to the previous results reinforcing that the disease-free survival time of HCC patients in the high expression group (highly expressed HOXB-AS1) was significantly shorter (*p* = 0.0435) than those in the low expression group (Figure 2b).

### 3.3 Low expression of HOXB-AS1 inhibited cell proliferation, invasiveness, and migration

**Figure 3:**
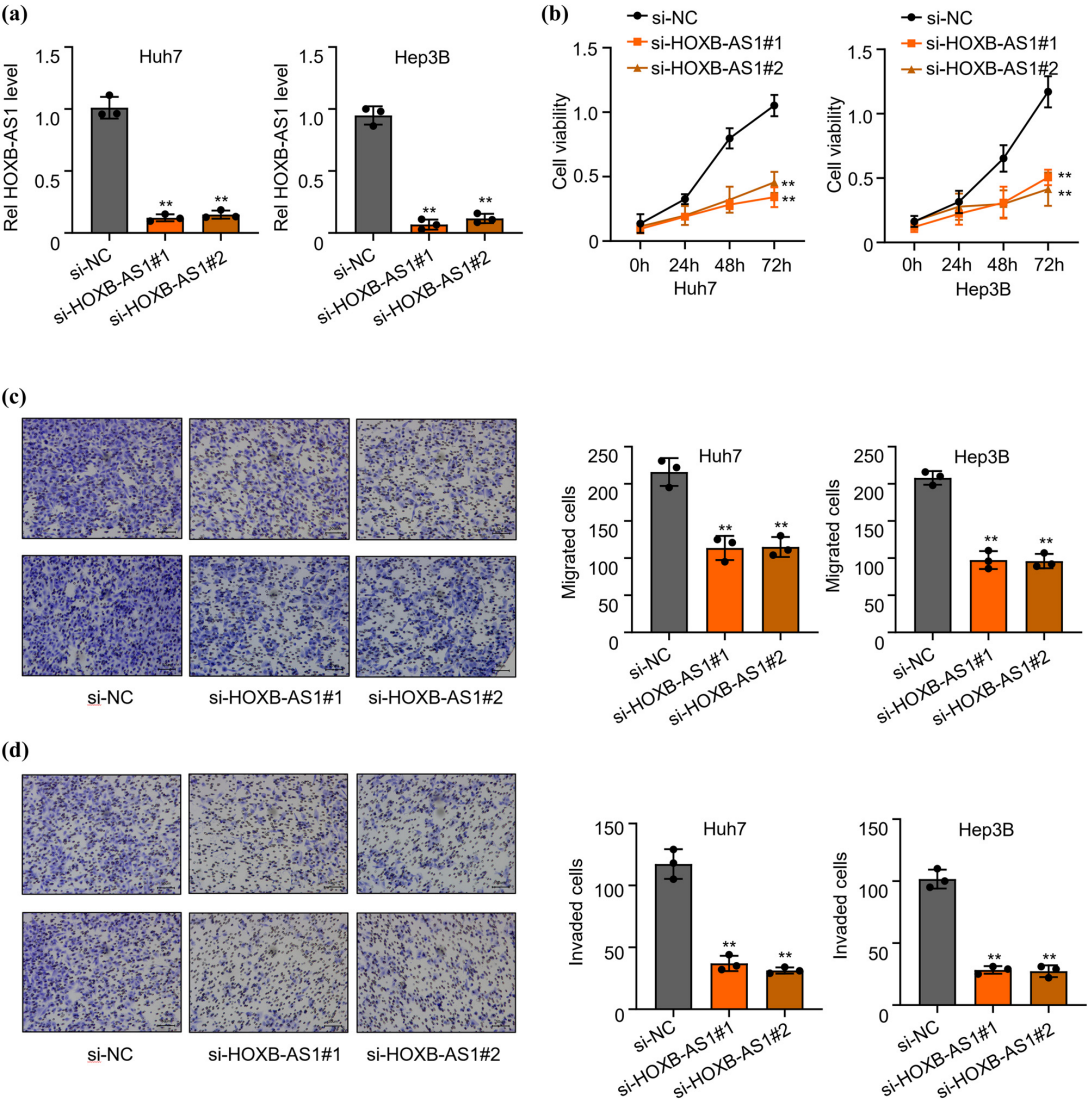
Low expression of HOXB-AS1 inhibited cell proliferation, invasiveness, and migration. (a) Silencing of HOXB-AS1 resulted in lower HOXB-AS1 expression, (b) cell viability, (c) migration, and (d) invasion of Huh7 and Hep3B cells transfected with si-HOXB-AS1#1 and si-HOXB-AS1#2 detected through CCK-8 method. The results are representative of 3 separate experiments. **p < 0.01 vs si-NC group.

After confirming the role of HOXB-AS1 in HCC development, it was further assessed for its proliferative, invasive, and migratory potential. To analyze the significance of HOXB-AS1 in HCC development, qRT-PCR was carried out to detect the effectiveness of HOXB-AS1 knockdown in Hep3B and Huh7 cells. Results demonstrated that relative expression of HOXB-AS1 significantly decreased (**p < 0.01) in both cell lines after transfection with si-HOXB-AS1#1 and si-HOXB-AS1#2 as compared to the negative control (si-NC) group (Figure 3a).

Cell viability was confirmed through the CCK-8 method by monitoring the light absorption values at 0, 24, 48, and 72 h in both Hep3B and Huh7 cells. Results from the bioassay indicated that transfection with small interfering RNAs, si-HOXB-AS1#1 and si-HOXB-AS1#2, significantly impacted the light absorption ability of the cells, and HOXB-AS1 knockdown markedly decreased the cell viability of both the cells (Figure 3b). The results were further confirmed by the Transwell test (without matrix adhesive EMC) to confirm the cellular migration of HOXB-AS1 knockouts. The data, in consistence with previous experiments, showed that knockdown of HOXB-AS1 with si-HOXB-AS1#1 and si-HOXB-AS1#2 significantly (**p < 0.01) decreased cell migration of both the Hep3B and Huh7 cells in comparison to si-NC (negative control group) as shown in Figure 3c. A subsequent Transwell test (matrix adhesive EMC) to check the invasiveness of the Hep3B and Huh7 cells was performed. Again, the results reinforced the previous observation that small interfering knockouts of HOXB-AS1 (si-HOXB-AS1#1 and si-HOXB-AS1#2) significantly (**p < 0.01) reduced the invasive ability of the transfected Hep3B and Huh7 cells when compared to the negative control (Figure 3d).

### 3.4 Upregulated serum HOXB-AS1 is tumor-derived and acts as a potential diagnostic biomarker for HCC

**Figure 4:**
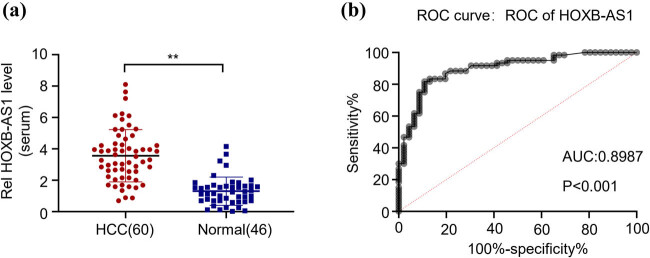
Upregulated serum HOXB-AS1 is tumor-derived and acts as a potential diagnostic biomarker for HCC. (a) Relative expression of serum HOXB-AS1 in 60 HCC patients and 46 normal healthy individuals. (b) ROC curve analysis to determine the sensitivity and specificity of the HOXB-AS1 serum test as a potential diagnostic tool. **p < 0.01 vs normal tissue.

After determining the oncogenic potential of HOXB-AS1 in HCC, the results were further subjected to serum analysis of HCC patients. When characterized by the relative expression levels of HOXB-AS1 in serum through qRT-PCR, the data indicated significantly elevated levels (**p < 0.01) of HOXB-AS1 in the serum of 60 HCC patients as compared to 46 normal individuals (Figure 4a). This suggests HOXB-AS1 as the potential prognostic and diagnostic biomarker of HCC patients. Finally, the ROC curve was utilized to analyze the diagnostic correctness and efficiency of serum HOXB-AS1. The data suggest high sensitivity and efficacy of serum diagnosis of HCC showing AUC > 0.7 (Figure 4d).

## 4 Discussion

Mounting research reports have highlighted the significance of lncRNAs, their aberrant expressions, elevated levels, and tumor inhibitor and promoter roles in several carcinomas [21,22]. By directly interfering with RNA, DNA, and proteins [14,15,16], the regulatory effects of several ncRNAs in many different groups of liver carcinogenesis are been recorded. For instance, lncRNA HOTTIP/HOXA13 expression is correlated with disease progression in HCC patients [23]. LncRNA PCNA-AS1 is an antisense RNA and regulates the proliferation of cellular nuclear antigens [24]. RNA HNF1A-AS1 has been recognized as an autophagy promotor that sponges hsa-miR-30b-5p and acts as an oncogene in HCC [15]. Similarly, the significance of HOXB-AS1 has been explained as an oncogene in several tumors. For example, HOXB-AS1 has been evaluated for its upregulated levels in glioblastoma cells and is directly associated with patient’s survival [25]. Elevated expression levels of HOXB-AS1 have been reported in myeloma and endometrial cancer [26,27]. However, its potential role in the proliferation and invasiveness of HCC is yet unknown. Thus, for the first time, our study revealed that HOXB-AS1 was significantly upregulated in HCC tissues, suggesting that it may be a key regulated gene with proliferative potential in HCC progression. Furthermore, knockdown of HOXB-AS1 reduces the proliferation of Hep3B and Huh7 cells and weakens their migration and invasion capabilities. This phenomenon is well-documented that suppression of several oncogenes’ lncRNAs significantly decreases the invasiveness, proliferation, and migration of tumorous cells. For instance, silencing of HOXB-AS1, HOXB2, or HOXB3 can lead to inhibition of cell proliferation and apoptosis stimulation of glioblastoma [17]. Similarly, silencing of HOXB-AS1 in myeloma cells suppressed myeloma cells’ proliferation and invasiveness [26]. Most importantly, this study showed the potential of serum analysis to detect HCC tumors by determining HOXB-AS1 levels in patient’s serum. When HOXB-AS1 levels were reported, the serum of 60 HCC patients showed considerably higher HOXB-AS1 expression as compared to 46 normal individuals indicating it as a promising prognostic biomarker. Further, serum test efficiency was determined by ROC curve analysis which showed its high accuracy and sensitivity in early detection of HCC tumors. However, our article has some limitations that need to be explored indepth in future. For example, we will explore the downstream regulatory mechanism of HOXB-AS1, including its effect on cell phenotype. In addition, in the next study, we will consider adding animal experiments to make the clinical significance of HOXB-AS1 more credible.

This study greatly expands our knowledge of HOXB-AS1 in regulating HCC progression function. We provide the first evidence that HOXB-AS1 functions as a critical player in regulating proliferation, migration, and invasion in Hep3B and Huh7 cells, laying the foundation for further clarifying the roles of HOXB-AS1 in HCC development. The results also highlighted HOXB-AS1 as a promising biomarker for the early diagnosis and prognosis of patients with HCC.

## References

[1] Cervello M, McCubrey JA, Cusimano A, Lampiasi N, Azzolina A, Montalto G. Targeted therapy for hepatocellular carcinoma: novel agents on the horizon. Oncotarget. 2012;3(3):236–60. 10.18632/oncotarget.466 PMC3359882 22470194

[2] El–Serag HB, Rudolph KL. Hepatocellular carcinoma: epidemiology and molecular carcinogenesis. Gastroenterology. 2007;132(7):2557–76. 10.1053/j.gastro.2007.04.061 17570226

[3] Rosenbloom KR, Dreszer TR, Long JC, Malladi VS, Sloan CA, Raney BJ, et al. ENCODE whole-genome data in the UCSC Genome Browser: update 2012. Nucleic Acids Res. 2012;40(D1):D912–7. 10.1093/nar/gkr1012 PMC3245183 22075998

[4] Qureshi IA, Mattick JS, Mehler MF. Long non-coding RNAs in nervous system function and disease. Brain Res. 2010;1338:20–35. 10.1016/j.brainres.2010.03.110 PMC2883659 20380817

[5] Wilusz JE, Sunwoo H, Spector DL. Long noncoding RNAs: functional surprises from the RNA world. Genes Dev. 2009;23(13):1494–504. 10.1101/gad.1800909 PMC3152381 19571179

[6] Quinn JJ, Chang HY. Unique features of long non-coding RNA biogenesis and function. Nat Rev Genet. 2016;17(1):47–62. 10.1038/nrg.2015.10 26666209

[7] Ward M, McEwan C, Mills JD, Janitz M. Conservation and tissue-specific transcription patterns of long noncoding RNAs. J Hum Transcriptome. 2015;1(1):2–9. 10.3109/23324015.2015.1077591 PMC4894084 27335896

[8] Huang Jf, Guo YJ, Zhao CX, Yuan SX, Wang Y, Tang GN, et al. Hepatitis B virus X protein (HBx)‐related long noncoding RNA (lncRNA) down‐regulated expression by HBx (Dreh) inhibits hepatocellular carcinoma metastasis by targeting the intermediate filament protein vimentin. Hepatology. 2013;57(5):1882–92. 10.1002/hep.26195 23239537

[9] Tsang FH, Au SL, Wei L, Fan DN, Lee JM, Wong CC, et al. Long non‐coding RNA HOTTIP is frequently upregulated in hepatocellular carcinoma and is targeted by tumour suppressive miR‐125b. Liver Int. 2015;35(5):1597–606. 10.1111/liv.12746 25424744

[10] Wang J, Liu X, Wu H, Ni P, Gu Z, Qiao Y, et al. CREB upregulates long non-coding RNA, HULC expression through interaction with microRNA-372 in liver cancer. Nucleic Acids Res. 2010;38(16):5366–83. 10.1093/nar/gkq285 PMC2938198 20423907

[11] Huang J-J, Ma SX, Hou X, Wang Z, Zeng YD, Qin T, et al. Toxic epidermal necrolysis related to AP (pemetrexed plus cisplatin) and gefitinib combination therapy in a patient with metastatic non-small cell lung cancer. Chin J Cancer. 2015;34(2):94–8. 10.5732/cjc.014.10151 PMC4360078 25418188

[12] Shukla R, Upton KR, Muñoz-Lopez M, Gerhardt DJ, Fisher ME, Nguyen T, et al. Endogenous retrotransposition activates oncogenic pathways in hepatocellular carcinoma. Cell. 2013;153(1):101–11. 10.1016/j.cell.2013.02.032 PMC3898742 23540693

[13] Kogo R, Shimamura T, Mimori K, Kawahara K, Imoto S, Sudo T, et al. Long noncoding RNA HOTAIR regulates polycomb-dependent chromatin modification and is associated with poor prognosis in colorectal cancers. Cancer Res. 2011;71(20):6320–6. 10.1158/0008-5472.CAN-11-1021 21862635

[14] Li T, Xie J, Shen C, Cheng D, Shi Y, Wu Z, et al. Amplification of long noncoding RNA ZFAS1 promotes metastasis in hepatocellular carcinoma. Cancer Res. 2015;75(15):3181–91. 10.1158/0008-5472.CAN-14-3721 26069248

[15] Liu Z, Wei X, Zhang A, Li C, Bai J, Dong J. Long non-coding RNA HNF1A-AS1 functioned as an oncogene and autophagy promoter in hepatocellular carcinoma through sponging hsa-miR-30b-5p. Biochem Biophys Res Commun. 2016;473(4):1268–75. 10.1016/j.bbrc.2016.04.054 27084450

[16] Salzman J. Circular RNA expression: its potential regulation and function. Trends Genet. 2016;32(5):309–16. 10.1016/j.tig.2016.03.002 PMC4948998 27050930

[17] Bi Y, Mao Y, Su Z, Du J, Ye L, Xu F. HOXB‐AS1 accelerates the tumorigenesis of glioblastoma via modulation of HOBX2 and HOBX3 at transcriptional and posttranscriptional levels. J Cell Physiol. 2020;236(1):93–106. 10.1002/jcp.29499 33459377

[18] Sun Y, Pan J, Zhang N, Wei W, Yu S, Ai L. Knockdown of long non-coding RNA H19 inhibits multiple myeloma cell growth via NF-κB pathway. Sci Rep. 2017;7(1):1–10. 10.1038/s41598-017-18056-9 PMC5741752 29273733

[19] Schefe JH, Lehmann KE, Buschmann IR, Unger T, Funke-Kaiser H. Quantitative real-time RT-PCR data analysis: current concepts and the novel “gene expression’s CT difference” formula. J Mol Med. 2006;84(11):901–10. 10.1007/s00109-006-0097-6 16972087

[20] Zhang H, Yan T, Liu Z, Wang J, Lu Y, Li D, et al. MicroRNA‐137 is negatively associated with clinical outcome and regulates tumor development through EZH2 in cervical cancer. J Cell Biochem. 2018;119(1):938–47. 10.1002/jcb.26259 28681918

[21] He M, Yang H, Shi H, Hu Y, Chang C, Liu S, et al. Sunitinib increases the cancer stem cells and vasculogenic mimicry formation via modulating the lncRNA-ECVSR/ERbeta/Hif2-alpha signaling. Cancer Lett. 2022;524:15–28. 10.1016/j.canlet.2021.08.028 34461182

[22] Zhang H, Ma RR, Zhang G, Dong Y, Duan M, Sun Y, et al. Long noncoding RNA lnc-LEMGC combines with DNA-PKcs to suppress gastric cancer metastasis. Cancer Lett. 2022;524:82–90. 10.1016/j.canlet.2021.09.042 34626692

[23] Quagliata L, Matter MS, Piscuoglio S, Arabi L, Ruiz C, Procino A, et al. Long noncoding RNA HOTTIP/HOXA13 expression is associated with disease progression and predicts outcome in hepatocellular carcinoma patients. Hepatology. 2014;59(3):911–23. 10.1002/hep.26740 PMC3943759 24114970

[24] Yuan S-X, Tao QF, Wang J, Yang F, Liu L, Wang LL, et al. Antisense long non-coding RNA PCNA-AS1 promotes tumor growth by regulating proliferating cell nuclear antigen in hepatocellular carcinoma. Cancer Lett. 2014;349(1):87–94. 10.1016/j.canlet.2014.03.029 24704293

[25] Chen X, Li LQ, Qiu X, Wu H. Long non-coding RNA HOXB-AS1 promotes proliferation, migration and invasion of glioblastoma cells via HOXB-AS1/miR-885-3p/HOXB2 axis. Neoplasma. 2019;66:2019–396. 10.4149/neo_2018_180606N377 30784279

[26] Chen R, Zhang X, Wang C. LncRNA HOXB‐AS1 promotes cell growth in multiple myeloma via FUT4 mRNA stability by ELAVL1. J Cell Biochem. 2020;121:4043–51. 10.1002/jcb.29573 31886581

[27] Liu D, Qiu M, Jiang L, Liu K. Long Noncoding RNA HOXB-AS1 Is Upregulated in Endometrial Carcinoma and Sponged miR-149-3p to Upregulate Wnt10b. Technol cancer Res & Treat. 2020;19:1533033820967462. 10.1177/1533033820967462 PMC7592328 33073693

